# Deciphering the star codings: astrocyte manipulation alters mouse behavior

**DOI:** 10.1038/s12276-020-0468-z

**Published:** 2020-07-15

**Authors:** Keebum Park, Sung Joong Lee

**Affiliations:** 1 Interdisciplinary Program in Neuroscience, College of Natural Sciences, Seoul National University, Seoul, Republic of Korea; 2 Department of Physiology and Neuroscience, Dental Research Institute, Seoul National University School of Dentistry, Seoul, Republic of Korea

**Keywords:** Astrocyte, Biological techniques

## Abstract

Astrocytes occupy a vast area within the central nervous system (CNS). Despite their abundance, the functional role of astrocytes in vivo has only begun to be uncovered. Astrocytes were typically thought to be involved in pathophysiological states. However, recent studies have shown that astrocytes are actively involved in cell signaling in normal physiological states; manipulating various aspects of astrocytic cell signaling in vivo has revealed that astrocytes are key players in controlling healthy behavior in the absence of pathophysiology. Unfortunately, the study of astrocyte function is often limited by the number of approaches available due to our lack of understanding of cell physiology. This review summarizes recent studies in which altered astrocyte signaling capacity resulted in dramatic changes in behavior. We not only discuss the methodologies available to manipulate astrocytes but also provide insights into the behavioral roles of astrocytes in the CNS.

## Animal behavior: Starring roles for star-shaped cells

Genetic studies provide increased evidence that astrocytes, star-shaped cells in the central nervous system, play important roles affecting behavior in mammals. Although they are just as abundant as neurons, astrocytes are not excited by electrical signals. For this reason they have traditionally been regarded simply as ‘support cells’ for neurons, but recent evidence suggests that they can significantly modulate neuron signals. A review paper by Keebum Park and Sung Joong Lee at Seoul National University in South Korea highlights improved methods for monitoring the signaling processes related to astrocytes, which manifest most notably through sharp changes in calcium levels. Several studies have used genetic knockout mice, designer drugs and light-sensitive proteins to change astrocyte activity, affecting a diverse range of behaviors including sleeping and feeding patterns, memory formation, depression and obsessive compulsive disorder.

## Introduction

Astrocytes, or “star-shaped” cells, which are as abundant in our CNS as neurons, densely and homogeneously populate the brain, spinal cord, and retina^1,2^. Although astrocytes are abundantly distributed across the entire brain, they have traditionally been regarded as “support cells” that play metabolic and homeostatic roles for nearby neurons^3^. The first evidence that neurons and astrocytes exchange functional signaling was presented ~30 years ago^4–6^. Later studies found that the star-shaped cells are particularly active in processing pain and inflammation^7–9^. Since then, studies have mainly focused on the role of astrocytes within the context of the brain’s pathophysiological states^10,11^. However, their role in physiological brain function in vivo has not been investigated for a while.

Over the last decade, there has been accumulating evidence that astrocytes actively participate in the brain’s physiological activities. Recent studies have also recognized the importance of the functional and regulatory roles that astrocytes play under normal physiological conditions. The final outputs that produce one’s behavior are generally accepted to be neuronal; however, astrocytes often critically modulate this final output. This relationship requires dynamic cell signaling between neurons and astrocytes. Morphologically, astrocytes have numerous processes that are in close proximity to neurons, other astrocytes, and blood vessels^12^. Astrocytic processes make contact with neuronal synapses and form tripartite synapses. A single astrocyte can make as many as 140,000 contacts with neuronal synapses^13^. Astrocytes actively exchange signals with nearby cells to complement and modulate neuronal communications in their vicinity. They often alter the electrophysiological properties of the surrounding area^14^. Therefore, it would not be unexpected for some of the astrocyte’s modulatory effects to influence behavioral outcomes in vivo. The hypothesis that astrocytes are directly involved in brain activity is further supported by recent studies showing how these cells are transcriptionally, translationally, morphologically, and functionally diverse across brain regions^15^. This evidence is important for understanding the more active roles of astrocytes under various physiological states. Efforts to uncover the effects of astrocytes have yielded valuable insights and tools for researchers. Consequently, astrocytes are no longer regarded as passive auxiliary support cells that function uniformly throughout the CNS.

Despite such advances and endeavors, however, a large portion of astrocyte physiology remains vague, and it is unclear how astrocytes process information to affect behavior^16^. Therefore, studies are needed to characterize astrocyte functions as well as their roles in the modulation of various behaviors. In this review, we highlight recent studies in which artificial disruption or enhancement of astrocyte cell signaling capacity in vivo resulted in immediate behavioral changes. These studies used experimental designs including but not limited to pharmacological, genetic, optogenetic, and chemogenetic approaches to specifically target astrocyte signaling processes. Additionally, we summarize the evidence for and provide current perspectives on the roles that astrocytes play in cell signaling and their critical impacts on healthy behaviors in rodents.

## Astrocytic cell signaling

Manipulating astrocytes requires a profound understanding of their signaling processes because each step may be important in understanding astrocyte function. Astrocytes are not electrically excitable cells; nevertheless, they express wide range of functional neurotransmitter receptors^17^. One of the most prominent responses that astrocytes have to an external input is elevating intracellular Ca^2+^ levels. Following the first report of astrocytic Ca^2+^ elevation^18^, there have been multiple attempts to understand the dynamics, origins, and implications of this process. These attempts have included the use of fluorescent Ca^2+^ indicator dyes, such as fura-2, rhod-2, or fluo-4, and genetically encoded calcium indicators (GECIs), such as the calmodulin-containing GCaMP series. Fluorescent Ca^2+^ indicators have been used to uncover several important physiological properties of astrocytes^19,20^ and are still the primarily used indicators in in vitro studies. However, some studies have reported difficulties in maintaining uniform levels of the dyes across the entire cell over an extended period of time^21^. These dyes have also been reported to be toxic to cells^22^. Given the clear limitations to this approach, most in vivo studies use GECIs to measure Ca^2+^ elevations in astrocytes. GECIs are stably expressed over time and do not alter normal signaling levels or have detrimental effects on cells; therefore, they are particularly suitable for long-term in vivo recordings^23^. As the use of GCaMPs has become the standard calcium-measuring method for both neurons and astrocytes, the series has been continually adjusted to improve the resolution, localization, and stability^24,25^. While traditional GECIs allowed researchers to only observe a single Ca^2+^ dynamics, recent technological advancements have enabled multilayered approaches to imaging and measuring Ca^2+^ dynamics. Modified GECIs^24^ keep similar wavelengths from interfering with one another and are less sensitive to artificial stimuli such as light. Researchers are also able to mark neurons and astrocytes simultaneously using multicolored GECIs^25^ to deduce temporal associations between regional cell types.

Astrocytic Ca^2+^ arises from multiple sources. A major source of Ca^2+^ elevation is the endoplasmic reticulum, in which Ca^2+^ release is triggered via activated inositol triphosphate (IP_3_) receptors^26^. Interestingly, both Gq-coupled and Gi-coupled G-protein coupled receptors (GPCRs) have been shown to elevate Ca^2+^ levels in astrocytes^27–29^. While several studies that targeted the GPCR-IP_3_ pathway to control astrocytic Ca^2+^ activity have failed to identify any noticeable physiological effects^30,31^, other studies performed with Ip3r2^−/−^ mice have found that this receptor is mainly responsible for somatic Ca^2+^ signals but not for Ca^2+^ signaling in cell processes^32^. This implies that there are other factors contributing to Ca^2+^ elevation in astrocytes, particularly in cell processes. Furthermore, the presence of spontaneous and seemingly random patches of Ca^2+^ in astrocytes is mediated by ion influx through transient receptor potential (TRP) A1 and V1 channels^33,34^. The TRPA1 channel is also responsible for maintaining resting Ca^2+^ levels^33^. More recently, it was shown that mitochondria mediate localized Ca^2+^ transients in astrocyte microdomains^35^. Taken together, these findings demonstrate that astrocyte Ca^2+^ signaling is highly dynamic. Assessing the implications of astrocyte function requires analysis with more spatiotemporal specificity, as well as comparisons of spontaneous and evoked, fast and slow, and global and focal signaling^36^. Nevertheless, studies that address the sources of astrocytic Ca^2+^ provide important information on potential targets for manipulating the astrocytic Ca^2+^ response.

After astrocytes receive signals and internally process them (primarily through Ca^2+^ elevation), they often emit signals to nearby cells. There is strong evidence that astrocytes are able to release signaling substances^16^ and that strong Ca^2+^ elevation precedes gliotransmitter release^36^. Gliotransmitters are neuroactive molecules released from astrocytes. These include glutamate, d-serine, ATP, and more. Upon their release into extracellular medium, gliotransmitters can exert an influence on local neurotransmission^37^. Findings suggest that astrocytes utilize SNARE-mediated vesicular exocytosis mechanisms for gliotransmission. Electron microscopy studies have detected gliotransmitters in labeled astrocytic vesicles^38^. In addition, soluble NSF attachment protein receptor (SNARE) isoforms have been identified in astrocytes, which also supports the vesicular-release model^39^. While the Ca^2+^ dynamics in astrocytes seem mainly asynchronous and oscillatory, one study demonstrated that gliotransmitter release is much more strongly correlated with a single large calcium elevation than with multiple small oscillatory waves^40^. Astrocytes have been shown to release d-serine to modulate long-term potentiation (LTP) in the CA1 region of the hippocampus^41^. They also release glutamate to induce timing-dependent long-term depression in the somatosensory cortex^42^ and release adenosine triphosphate (ATP) in response to mechanical stretch in the hippocampus^43^. It is not yet known whether a single astrocyte can release multiple neurotransmitters simultaneously or whether these cells can distinguish different input signals by differentiating their output transmitters. It also remains to be determined whether the release mechanism for each gliotransmitter is unique^44^. When astrocytes become reactive after injury or inflammation, their morphology and Ca^2+^ signaling patterns change drastically. Following a pathophysiological state in the brain, the amplitude and frequency of Ca^2+^ signals generally tend to increase^43^. In some models, astrocytic signals occur earlier than neuronal signals^38^. Although the detailed mechanism through which reactive astrocytes display heightened signaling is not completely understood, there is evidence that the aforementioned IP_3_ and TRPA1 pathways contribute in some way^43^. Such signaling complexities provide multiple areas of possible investigation to elucidate the consequences of astrocyte functions.

**Fig. 1:**
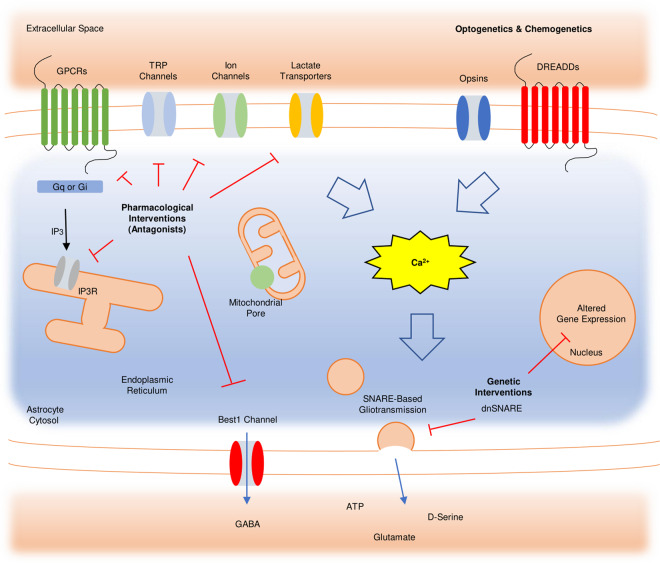
Astrocyte signaling pathways and intervention points. This schematic outlines some of the major signaling pathways in astrocytes. Changes in astrocytic intracellular Ca^2+^ levels depend on a number of activities by receptors, channels, pores, and other cellular components. Therefore, each component or process illustrated in this figure is also a possible intervention point to control intracellular Ca^2+^ levels. The components are roughly classified into several categories. Manipulating the activity of membrane proteins can be done using antagonists and blockers. GPCRs include mAChR, GABA_B_, and NMDAR, and the GPCR-IP_3_ pathway can also be regulated by controlling IP_3_R_2_ activity. Other membrane proteins, such as TRP channels, ion channels, and voltage-gated calcium channels (not shown), are also responsible for the Ca^2+^ response in astrocytes. Elevated astrocyte Ca^2+^ often leads to altered gene expression and SNARE-based gliotransmission. Although the gliotransmitter released varies between brain regions, dominant-negative SNARE proteins can be utilized to restrict gliotransmission.

The approaches that are discussed in this review either enhance or impair one of the signaling pathways described above (Fig. 1). In genetic knockout models, such as Ip3r2^−/−^ mice and Trpa1^−/−^ mice, some of the known contributors to Ca^2+^ elevation in astrocytes are eliminated. Other studies genetically have introduced artificial pumps in astrocyte membranes to eliminate all intracellular calcium ions^45^. SNARE activity can also be modulated to regulate gliotransmission. Manipulation of designer receptors exclusively activated by designer drugs (DREADDs) has also been employed. These nonendogenous receptors are coupled with either Gq(hM3Dq) or Gi(hM4Di) to modulate astrocyte activity. However, there are reports that the Ca^2+^ levels in astrocytes increase in response to both Gq and Gi^28,29^, albeit to different degrees, and the specific differences between the two have not yet been characterized in vivo. Light-sensitive channelrhodopsin family proteins have also been used to study astrocytes. Although astrocytes are not electrically excitable, cation channels greatly increase Ca^2+^ levels in astrocytes^46^. Recently, the G-protein-coupled photopigment melanopsin was used for precise optogenetic astrocyte activation^47^. Admittedly, some of the methods employed by recent studies, especially optogenetic manipulation, have been brought into question^48,49^. As our understanding of the intercellular signals in astrocytes improves, there will be more layers to explore beyond those able to be studied with the currently available investigative approaches.

## Astrocyte manipulation in vivo

### Astrocytes in circadian rhythm and sleep

One of the first behaviors that is suspected to be under astrocyte control is circadian rhythm, in part because the earliest understanding of astrocytes suggested that they regulate homeostatic control. The mammalian circadian system is derived from oscillatory regulation of transcription factors. It is imperative for organisms to maintain this timekeeping system to maintain concordance with the daily cycle. The circadian rhythm is thought to be supervised by neurons in the suprachiasmatic nucleus (SCN). These neurons express the transcription factors brain and muscle Arnt-like protein-1 (BMAL1) and circadian locomotor output cycles kaput (CLOCK) in a feedback loop fashion^50^. These neurons have been the focus of many circadian rhythm studies, while astrocytes have largely been overlooked. However, there is clear evidence that astrocytes rhythmically express circadian oscillators such as *Bmal1* as well^51^. Astrocytes are also able to receive circadian inputs from nearby SCN neurons and release gliotransmitters in response^51^. It was uncertain whether astrocyte rhythmic activity led to circadian behavior until the recent work by Brancaccio et al.^52^. They used cryptochrome circadian regulator 1/2 (Cry1/2)-null mice, which are circadian-incompetent. Since CRY1 and CRY2 are necessary for a transcription–translation negative feedback loop (TTFL), Cry1/2-null mice have dysfunctional TTFL cycles and display no circadian behavior^52^. *Cry1* was then selectively restored in SCN astrocytes or neurons by injecting Cre-dependent AAVs encoding *Cry1::EGFP* with GFAP-Cre or Syn-Cre AAV constructs. As expected, complementing *Cry1* in SCN neurons quickly restored the TTFL, and the mice began exhibiting ~26-h behavior cycles^52^. Interestingly, complementing *Cry1* only in the SCN astrocytes of Cry1/2-null mice was sufficient to induce the TTFL and restore circadian behavior^52^. This effect was largely blocked by inhibiting Cx43, an astrocyte-specific connexin hemichannel that is responsible for gliotransmitter release. In addition, treatment with the NMDA receptor antagonist DQP-1105 also blocked the rescue effect. These results suggest that astrocytic glutamate signaling is responsible for the restoration of circadian behavior^52^. In accordance with this, Tso et al.^53^ also focused on the function of SCN astrocytes in circadian signaling by specifically knocking out the circadian oscillator gene *Bmal1* in SCN astrocytes using clustered regularly interspaced short palindromic repeats (CRISPR) with CRISPR-associated protein 9 (Cas9). Although astrocytes were shown to be functional circadian oscillators under normal conditions, the loss of *Bmal1* disrupted the TTFL in astrocytes and significantly lengthened the circadian period^53^. Similarly, the presence of the casein kinase 1 epsilon (CK1ε) *tau* mutation specifically in astrocytes (Aldh1L1-CK1ε^tau/+^) shortened the circadian period. Restoring this mutation in the SCN area somewhat rescued the phenotype^53^. This study did not specifically describe how astrocytes control the circadian rhythm; however, Brancaccio et al. predicted that it is partially due to a disruption in astrocyte-neuron signaling in the SCN^52^. Such an interaction also appears to occur in other species, as the astrocyte-specific expression of the circadian factor *Ebony* rescued circadian behaviors in *Drosophila*^54^. These studies suggest that manipulating astrocyte signaling in the SCN can drastically modulate circadian locomotion. It is especially notable that astrocytic circadian control alone is sufficient to drive circadian behavior and that dysfunctional astrocytic circadian control alone is sufficient to break circadian behavior. In both cases, astrocytic outputs completely overshadow the effect of neuronal outputs. These results suggest that astrocytes are not just complementary to neurons but instead are some of the most important players in controlling circadian behaviors.

Beyond circadian locomotive behaviors, several studies have suggested that controlling astrocyte signaling also affects sleeping patterns. Sleep homeostasis is maintained by adenosine accumulation, which is why caffeine, an adenosine receptor antagonist, promotes wakefulness^55^. The source of this adenosine was assumed to be neuronal. However, several studies have clearly demonstrated that this adenosine comes from astrocytes. Not surprisingly, one of the most well-known gliotransmitters is ATP. Astrocytic exocytosis via the SNARE complex can be disturbed by introducing a dominant-negative version (dnSNARE), resulting in impaired gliotransmission. When astrocytic dnSNARE was systematically expressed in mice, sleep homeostasis was impaired, and cortical electroencephalogram (EEG) recordings showed attenuated slow wave activity following sleep deprivation^56^. An increase in slow waves is a normal compensatory response to accumulating sleep pressure. The attenuated slow wave power was partially rescued when the A1 receptor antagonist cyclopentyltheophylline was applied intraventricularly, which indicates that purinergic gliotransmission contributes to sleep homeostasis^56^. Alternatively, when cortical astrocytes were optogenetically stimulated in vivo with channelrhodopsin-2 (ChR2), nearby neurons switched to slow wave oscillations^57^. Chemogenetic activation also obtained the same result^29^. It is also notable that astrocytes massively synchronize their Ca^2+^ activity before neurons drift into slow wave activity^58^. Another study dampened astrocytic Ca^2+^ activity to induce slow wave oscillations. Expressing the Venus-IPP fusion protein specifically in astrocytes enhanced IP_3_ to IP_2_ metabolism and attenuated agonist-induced Ca^2+^ signaling in astrocytes^59^. When this IP_3_/Ca^2+^ pathway was impaired in hippocampal astrocytes, mice exhibited elevated slow wave power and increased rapid-eye movement (REM) sleep duration without alterations in their non-REM (NREM) sleep pattern^59^. In addition, optogenetic stimulation of astrocytes in the hypothalamus at 10 Hz increased both NREM and REM sleep sessions within a 6-h period^60^. In *Drosophila*, the astrocytic release of a TNF-α homolog is necessary for a sleep deprivation-induced homeostatic response^61^. In that study, purinergic gliotransmission was responsible for the accumulation of sleep pressure. Interestingly, two different astrocytic agents drove seemingly opposing effects. Astrocytic glutamate promoted the physiological circadian rhythm, while astrocytic ATP promoted a wakeful state over a sleep state. However, the astrocytic dnSNARE models used in these studies restricts astrocytic gliotransmission globally. Although the in vitro data suggest that the hippocampus is the region responsible for promoting the wake state, this has not been confirmed in vivo. It is also interesting to note that purinergic gliotransmission is also responsible for negative effects that accompany disruption of the circadian cycle, such as impaired learning and memory, which will be further discussed in a later section.

### Astrocytes and cognition

Cognition, learning, and memory are some of the biggest mysteries in the field of neuroscience. Although significant progress has been made in understanding how we form and recall memories, numerous aspects of memory are yet to be elucidated. Several studies suggest that astrocytes contribute to this process, and acknowledging their roles would advance our understanding of cognitive processes in general. There is accumulating evidence that astrocytes are critically involved in memory formation through various mechanisms, such as by selectively strengthening synapses. Martin-Fernandez et al. used the endocannabinoid and DREADD systems to chemogenetically manipulate astrocytes in the medial subdivision of the central amygdala (CeM)^62^. Astrocytes in this region respond to endogenous cannabinoids via astrocytic cannabinoid receptor type 1 (CB1R) to have elevated Ca^2+^ levels. This then decreases the excitatory input from the basolateral amygdala (BLA) to the CeM^62^. Astrocytic expression of stimulatory Gq-DREADD hM3D in this area also causes elevated Ca^2+^ levels and similar neurophysiological changes. Therefore, both endocannabinoids and Gq DREADD activation of CeM astrocytes caused mice to be completely incapable of forming fear memories in a cued fear-conditioning task^62^. The study also found that elevated Ca^2+^ levels in astrocytes-triggered ATP release and modulated A1R/A2R activities to selectively enhance inhibitory synapses in the CeM^62^. In several other studies, an astrocytic dnSNARE mouse model was employed to show that gliotransmitters are involved in memory formation. Regulating astrocytic ATP release with dnSNARE alleviated post-sleep deprivation memory impairment in object recognition tests by rescuing synaptic plasticity in the hippocampus^56,63^. This implies that astrocytic ATP not only forces the awake state when one is deprived of sleep but also somewhat eliminates the negative consequences of sleep deprivation. Interestingly, these mice did not seem to accumulate sleep pressure, as if they did not require sleep. In addition to ATP, activity-dependent d-serine is absent in the hippocampus in astrocytic dnSNARE mice^64^. d-serine is an endogenous coagonist of NMDA receptors (NMDARs) at CA3-CA1 synapses. dnSNARE mice performed poorly in contextual fear memory, which was rescued by external d-serine administration^64^. More recent studies have also demonstrated that astrocytic d-serine release depends on astrocytic CB1 receptor activity. One genetic knockout model (GFAP-*CB*_*1*_-KO) showed reduced NMDAR active-site occupancy, reduced in vivo hippocampal LTP, and impaired object recognition memory^65^. Along with gene knockout, optogenetic and chemogenetic activation of CA1 astrocytic signaling was also sufficient to enhance memory formation in a context-dependent manner for contextual fear memory and the T-maze^66^. In that study, Opto-Gq and Gq-DREADD hM3D were employed to demonstrate that astrocytic stimulation is sufficient to promote LTP in the hippocampus. Although neuronal stimulation of the area impaired memory performance, astrocyte stimulation enhanced it. However, astrocytic memory enhancement was situational. When astrocyte stimulation occurred with learning itself, cognitive augmentation occurred^66^. The effects of NMDAR activity shown in that study imply that d-serine is involved in the de novo potentiation of CA3 to CA1 synapses^66^. By intervening in astrocyte cell signaling through various methods, multiple studies have found a correlation between astrocyte activity and memory formation. This relationship appears to involve gliotransmission, notably transmission involving ATP and d-serine, in the hippocampus. Multiple studies agree that astrocytic d-serine release occurs in the hippocampus as the Gq pathway is activated. This d-serine critically modulates LTP formation in this area. However, gliotransmission is dysfunctional in some pathophysiological models. In a mouse model of Alzheimer’s disease (AD), astrocytes release excessive GABA through the *Best1* channel. The cognitive symptoms exhibited by a mouse model of AD were alleviated by oral administration of selegilline, which blocks monoamine oxidase B activity, ultimately blocking astrocytic GABA synthesis^67^. Therefore, this study identified GABA as another potential gliomodulator of cognition. In vitro data suggest that hippocampal activities can be rescued by either inhibiting GABA synthesis or blocking the *Best1* channel. However, a GABA synthesis inhibitor was not administered in vivo in the above-mentioned study. Therefore, further confirmation of this phenomenon is necessary.

The studies that we have described thus far have concluded that astrocyte signaling results in gliotransmitter release that affects synaptic function. Another interesting hypothesis is that rather than modulating synapses, astrocytes modulate the activity of nearby neurons by supplying them with energy in the form of lactate. The significance of lactate transportation in the CNS was first described in 2011^68^. Since then, multiple groups have investigated the in vivo behavioral role of the lactate transport system in astrocytes. Glycolysis in astrocytes results in lactate release via monocarboxylate transporters (MCTs) 1 and 4 (astrocytes) to MCT2 (neurons). One study found that disruption of the astrocytic lactate transporters MCT1 and MCT4 in the hippocampus caused amnesia^68^. Adrenergic signaling of the area triggers astrocytic release of lactate, and β_2_-adrenergic receptors (β_2_AR) are expressed by hippocampal astrocytes. When exposed to a β_2_AR antagonist, mice failed to exhibit long-term inhibitory avoidance memory formation tasks^69^. This effect was rescued when lactate was supplied locally, indicating that these receptors are responsible for learning-evoked lactate release from astrocytes in the region^69^. In accordance with this result, another study administered a β_2_AR agonist by injection over multiple days. This intervention activated downstream signaling pathways, including pathways associated with lactate metabolism and transportation, and greatly improved the performance of the animals in the Morris water maze test^70^. In the hippocampus, astrocyte-neuron lactate transfer is important for the consolidation/reconsolidation of appetitive conditioning. Infusion of glycogen phosphorylase into the BLA of rats impaired astrocyte-neuron lactate transportation. This effect not only prevented rats from forming the new cocaine-based conditioned place preferences but also disturbed existing preferences^71^. These studies collectively suggest that, in response to external stimuli, astrocytes undergo glycolysis to produce lactate and release it to adjacent neurons. Astrocytic lactate plays a crucial role in forming and recalling memories across multiple brain regions, including the hippocampus and amygdala. Combined with previous descriptions, it appears that hippocampal astrocytes modulate learning on multiple levels. Inputs coming into the hippocampus are sensed by local astrocytes. These inputs activate the Gq pathway and lead to Ca^2+^ elevation. Astrocytes then oversee lactate transportation and local adrenergic and d-serine signaling, which promote neuronal activity and synaptic plasticity in the area. An increasing amount of in vivo evidence suggests that hippocampal astrocytes are important for learning and memory. This evidence underscores the importance of fully elucidating neuron–astrocyte communication to solve one of the biggest mysteries in neuroscience.

### Astrocytes and mood-associated behaviors

Soon after astrocytes were found to react to nearby neuronal activity, they were also seen to respond rapidly to pathophysiological states in the brain. Traditionally, astrocytes were thought to mainly respond to physical damage in the brain, such as ischemia or neurodegenerative diseases (such as AD). Recent advancements in the understanding of astroglial functions suggest that astrocytes are also involved in neuropsychiatric pathophysiology. For instance, astrocytes display altered morphology when neuropathic pain is induced^43^. Similarly, major depressive disorder is associated with a reduced number and density of astrocytes, and astrocytes exhibit cell hypotrophy in both rodents and humans^72^. Cao et al.^73^ showed that astrocytic signaling can have both depressive and anti-depressive effects on mice. In this study, *Itpr2*^−*/*−^ transgenic mice were used to disrupt the IP_3_ signaling pathways in astrocytes. Astrocytic Ca^2+^ elevation and ATP release failed to be induced in knockout mice, but neuronal ATP release was unaffected^73^. These mice were susceptible to chronic social defeats and rapidly developed depression-like behaviors. Notably, systemic administration of ATP induced antidepressant-like effects in the mice^73^. In addition, the mice that were previously affected by chronic social defeat stress quickly recovered when astrocytic Gq signaling was enhanced by overexpression of the mas-related gene A1, which elevates intracellular Ca^2+^. These mice show decreased immobility in a forced swim test and increased interaction during social defeat sessions^73^. However, there is contradictory evidence regarding whether IP_3_R2-dependent Ca^2+^ signaling and gliotransmission induce anxiety or depressive-like behavior^74^. In the above-mentioned study, IP_3_R2 KO model mice did not show any abnormalities in the open field test or the tail suspension test. Therefore, given the multiple contradicting results, it remains difficult to clarify the relationship between astrocytic activity and depression. This controversy may be explained by the complex nature of depression and the lack of decisive behavior tests that are available to evaluate it.

In contrast, another mood-associated behavioral disorder is well known to be governed by astrocytes. Obsessive-compulsive disorder (OCD) is an anxiety disorder that is characterized by repetitive behaviors. The most prominent theory regarding the underlying mechanism of OCD is a dysregulated excitation to inhibition (E/I) ratio. As astrocytes are homeostatic controllers, several studies have found that astrocytes are involved in repetitive behavior. Astrocytes express glutamate transporter 1 (GLT1) to regulate the E/I balance. One study found that astrocyte-specific conditional GLT1 knockout mice (GLAST^CreERT2/+^/GLT1^flx/flx^) exhibited pathological levels of repetitive behaviors to the point of injuring themselves^75^. Systematic administration of NMDA antagonist alleviated this symptom, which suggests that astrocytic glutamate transportation via GLT1 plays a critical role in controlling repetitive behaviors^75^. In another study, a modified isoform of the human plasma membrane calcium pump (hPMCA2w/b) was exogenously expressed specifically in striatal astrocytes^45^. This calcium pump constitutively extrudes cytosolic Ca^2+^, which renders cells almost devoid of calcium. Although hPMCA2w/b mice did not display any anxiety or depression-like phenotypes, they demonstrated extensive self-grooming behavior when they were left alone^45^. As a putative underlying mechanism of such behavioral alterations, in vivo Ca^2+^ imaging revealed that medium spiny neuron (MSN) activity was significantly dampened when nearby astrocyte Ca^2+^ signaling was abolished. Blocking the astrocytic GABA transporter GAT-3 also partially rescued the phenotype. This result suggests that astrocytic Ca^2+^ signaling is responsible for tonic GABA inhibition in the striatum^40^. Interestingly, GABA release from MSNs results in acute behavioral hyperactivity and disrupted attention, which is rescued by inhibition of the astrocytic GABA_B_-Gi pathway^76^. Artificially activating this pathway also upregulates thrombospondin-1 in astrocytes, which in turn increases excitatory synapses and enhances cortical synaptic transmission^76^. These results suggest that MSNs and astrocytes in the striatum reciprocally exchange GABA signals. Additionally, tonic GABA signals that result from elevated astrocytic Ca^2+^ inhibit the activity of MSNs to produce repetitive behaviors (Table 1).

**Table 1:** In vivo manipulation of astrocyte signaling.

Behavior	Modulation	Tests	Major phenotype	Reference
Circadian rhythm	Selective restoration of astrocyte TTFL in Cry 1/2-null mice	Locomotion tracking	Restoration of circadian rhythm	^52^
	Selective disruption of astrocytic TTFL	Locomotion tracking	Lengthened circadian rhythm	^53^
	Disruption of astrocytic adenosine signaling via dnSNARE	Spatial object recognition	Resilience to sleep deprivation	^56^
	Disruption of astrocytic adenosine signaling via dnSNARE	EEG Novel object recognition	Decreased sleep pressure Resilience to sleep deprivation	^63^
	Optogenetic stimulation of cortical astrocytes via ChR2	EEG	Increased slow-wave activity in nearby neurons	^57^
	Chemogenetic stimulation of cortical astrocytes	Field recordings	Increased slow-wave activity in nearby neurons	^29^
	Attenuation of the IP_3_-induced Ca^2+^ response via Venus-IPP	EEG	Elevated sleep power Increased REM sleep duration	^59^
	Optogenetic stimulation of hypothalamus astrocytes	EEG	Increased REM and NREM sleep duration	^60^
Cognition	Activation of endogenous astrocytic endocannabinoid receptor in amygdala	Cued fear conditioning task	Increased intracellular Ca^2+^ Inability to form fear memories	^62^
	Chemogenetic stimulation of astrocytes in the amygdala	Cued fear conditioning task	Increased intracellular Ca^2+^ Inability to form fear memories	^62^
	Disruption of astrocytic adenosine signaling via dnSNARE	Spatial object recognition	Prevention of cognitive deficits following sleep deprivation	^56^
	Disruption of astrocytic adenosine signaling via dnSNARE	Novel object recognition	Prevention of cognitive deficits following sleep deprivation	^63^
	Disruption of astrocytic d-serine signaling via dnSNARE	Contextual fear memory test	Poor performance in a memory test	^64^
	Selective knockout of cannabinoid receptor	Object location test	Reduced hippocampal LTP Impaired object location memory	^65^
	Optogenetic stimulation of hippocampal astrocytes via Opto-Gq	Contextual fear memory test T-maze	Promotion of LTP in the hippocampus Enhanced memory formation	^66^
	Chemogenetic stimulation of hippocampal astrocytes via hM3Dq	Contextual fear memory test T-maze	Promotion of LTP in the hippocampus Enhanced memory formation	^66^
	Enhanced d-serine activity in hippocampal astrocytes	Contextual fear memory test	Promotion of LTP in the hippocampus Enhanced memory formation	^66^
Cognition	Inhibition of astrocytic GABA synthesis or GABA release in AD mice	Morris water maze	Improved LTP formation Improved memory formation	^67^
	Disruption of astrocytic lactate transportation in the hippocampus	Field recordings	Impaired hippocampal LTP, rescued by lactate	^68^
	Disruption of astrocytic lactate transportation in the hippocampus	Inhibitory avoidance test	Impaired long-term memory formation	^68^
	Disruption of astrocytic adrenergic signaling via AR antagonist	Inhibitory avoidance test	Impaired long-term memory formation, rescued by lactate	^69^
	Enhancement of astrocytic adrenergic signaling via AR agonist	Morris water maze	Improved memory performance	^70^
	Disruption of astrocytic lactate transportation in the BLA via glycogen phosphorylase	Reward-based conditioned place preference test	Inability to form new place preference Disruption of existing place preference	^71^
Mood-associated	Disruption of calcium signaling in astrocytes via Itpr2 KO	Two-photon microscopy	Failure to induce Ca^2+^ elevation Disruption of astrocytic ATP release	^73^
	Disruption of calcium signaling in astrocytes via Itpr2 KO	Chronic social stress	Development of depression-like behavior	^73^
	Enhancement of calcium signaling in astrocytes via Mas1 overexpression	Three-chamber test	Rescue of depression-like behavior	^73^
	Disruption of astrocytic glutamate transportation via selective GLT1 KO	Free-moving	Increased repetitive behavior	^75^
	Ablation of astrocytic calcium via selective expression of artificial calcium pump in striatum	Free-moving	Increased repetitive behavior	^45^
	Enhancement of the GABA_B_ pathway in striatal astrocytes	Field recordings Free-moving	Increased synaptic transmission Hyperactivity	^76^
Appetite	Chemogenetic activation of arcuate nucleus astrocytes via hM3Dq	Feeding test	Reduced ghrelin-evoked feeding behavior	^77^
	Chemogenetic inhibition of arcuate nucleus astrocytes via hM4Di	Feeding test	Increased and prolonged ghrelin-evoked feeding behavior	^77^
Approach-avoidance	Optogenetic activation of VTA astrocytes via ChR2	Real-time place preference test	Active avoidance of “light ON” chamber	^79^
	Disruption of astrocytic glutamate transport via GLT1 KO	Real-time place preference test	No preference under light stimulation	^79^
Motor skill	Disruption of calcium signaling in astrocytes via Ip3r2 KO	Motor skill learning	Decreased LTP in the motor cortex Poor performance in motor skill learning	^80^

### Other behaviors

Direct behavior control by astrocytes is not limited to the circadian rhythm, memory, and mood. Multiple studies have controlled various aspects of astrocyte signaling to produce diverse observable behaviors. Just as astrocytes control homeostasis of the circadian rhythm, they also regulate nutritional homeostasis. DREADD-mediated modulation of astrocytes in the arcuate nucleus using Gq-DREADD hM3D altered appetite in mice^77,78^. The mice demonstrated an acute increase in food consumption following CNO administration. Activated astrocytes also specifically interact with adjacent AgRP/NPY neurons via adenosine A_1_ receptors to modulate food intake^78^. Yang et al.^77^ found the opposite effect to be true when they “inhibited” astrocyte activity using Gi-DREADD hM4D. However, multiple other reports have found that the Gi pathway also results in elevated Ca^2+^ levels in astrocytes^15,28,29^. The Gq and Gi pathways result in different degrees of Ca^2+^ elevation^15^. However, it remains to be determined whether the Gi pathway truly is “inhibitory” and reverses the effects of the Gq pathway. In addition to feeding behavior, astrocytes are also involved in reward and aversive behavior. We have previously discussed a study showing how endocannabinoid input to astrocytes is necessary for reward-based learning^62^. Optogenetically stimulating astrocytes in the ventral tegmental area (VTA) with ChR2 resulted in clear, real-time avoidance of the “light-ON” area during a real-time place preference test^79^. Such avoidance behavior was absent in GLT1-cKO^VTA Astrocyte^ mice, indicating that astrocytic glutamate transport in the area is crucial for modulating aversive behavior^79^. In that study, astrocytes were shown to adjust the glutamatergic input to local GABA neurons that inhibit dopamine neurons in turn^79^. Although the brain subregions examined in these studies differed (the amygdala vs. the VTA), they both demonstrate that astrocytic signaling is crucial for the development of healthy approach-avoidance behavior. Another study suggested that proper motor skill learning depends on astrocyte signaling. When astrocyte Ca^2+^ signaling was attenuated via IP_3_R2 knockout or the astrocyte-specific metabolic inhibitor fluorocitrate, mice demonstrated poor performance in motor skill learning^80^. In that study, both IP_3_R2 deficiency and fluorocitrate treatment impaired LTP-like activity in the motor cortex. This impairment prevented motor skill training-induced glutamate transmission^80^. These findings indicate that astrocytic Ca^2+^ is implicated not only in decisive memory formation but also in procedural memory formation.

## Conclusion

The diverse behavioral roles of astrocytes in the CNS are becoming more apparent. In the last decade, astrocytes have emerged as critical modulators of information processing in the brain. Although numerous reports have been shifting attention from neurons to astrocytes, the precise in vivo functions of astrocytes have only begun to be decoded. This review discusses recent efforts aimed at elucidating how the astrocyte signaling process is carried out and presents wide-ranging evidence that controlling astrocyte activity can directly affect animal behaviors. Endeavors to decipher the star codings are relatively new, and our current understanding of astrocyte physiology is limited. Although assessing astrocyte function in behaviors in vivo is challenging, new technologies and more accurate models are constantly arising to help researchers precisely monitor and manipulate astrocyte activities in vivo, which will aid in establishing and validating more refined theories of behavioral neuroscience based on neuron–glia communication. The future of research on astrocytes in behavioral neuroscience promises to be fascinating and will likely accompany meaningful real-life applications in fields such as clinical medicine.
